# Toward Enhanced Teleoperation Through Embodiment

**DOI:** 10.3389/frobt.2020.00014

**Published:** 2020-02-11

**Authors:** Alexander Toet, Irene A. Kuling, Bouke N. Krom, Jan B. F. van Erp

**Affiliations:** ^1^Perceptual and Cognitive Systems, Netherlands Organisation for Applied Scientific Research (TNO), Soesterberg, Netherlands; ^2^Intelligent Autonomous Systems, Netherlands Organisation for Applied Scientific Research (TNO), The Hague, Netherlands; ^3^Human Media Interaction, University of Twente, Enschede, Netherlands

**Keywords:** teleoperation, body ownership illusion, rubber hand illusion, performance enhancement, embodiment, robotics, telemanipulation, robotic dexterity

## Abstract

Telerobotics aims to transfer human manipulation skills and dexterity over an arbitrary distance and at an arbitrary scale to a remote workplace. A telerobotic system that is transparent enables a natural and intuitive interaction. We postulate that embodiment (with three sub-components: sense of ownership, agency, and self-location) of the robotic system leads to optimal perceptual transparency and increases task performance. However, this has not yet been investigated directly. We reason along four premises and present findings from the literature that substantiate each of them: (1) the brain can embody non-bodily objects (e.g., robotic hands), (2) embodiment can be elicited with mediated sensorimotor interaction, (3) embodiment is robust against inconsistencies between the robotic system and the operator's body, and (4) embodiment positively correlates to dexterous task performance. We use the predictive encoding theory as a framework to interpret and discuss the results reported in the literature. Numerous previous studies have shown that it is possible to induce embodiment over a wide range of virtual and real extracorporeal objects (including artificial limbs, avatars, and android robots) through mediated sensorimotor interaction. Also, embodiment can occur for non-human morphologies including for elongated arms and a tail. In accordance with the predictive encoding theory, none of the sensory modalities is critical in establishing ownership, and discrepancies in multisensory signals do not necessarily lead to loss of embodiment. However, large discrepancies in terms of multisensory synchrony or visual likeness can prohibit embodiment from occurring. The literature provides less extensive support for the link between embodiment and (dexterous) task performance. However, data gathered with prosthetic hands do indicate a positive correlation. We conclude that all four premises are supported by direct or indirect evidence in the literature, suggesting that embodiment of a remote manipulator may improve dexterous performance in telerobotics. This warrants further implementation testing of embodiment in telerobotics. We formulate a first set of guidelines to apply embodiment in telerobotics and identify some important research topics.

## Introduction

### The Potential of Embodiment in Teleoperation

Despite the increasing availability of autonomous systems, robots that are remotely controlled by humans remain a key technology for operations, for instance in inaccessible areas (e.g., in space or deep-sea) or in complex, unpredictable or hazardous environments with a high degree of uncertainty, such as in minimally invasive surgery, search and rescue operations, disaster response or explosive ordnance disposal (Siciliano and Khatib, 2016). Telerobotics (Niemeyer et al., 2016) aims to replicate human manipulative skills and dexterity over an arbitrary distance and at an arbitrary scale to a remote workplace. Ideally, operators performing complex tasks with telemanipulators should have the impression of physically being at the point of interaction, so that the interaction itself feels natural and as intuitive as possible (referred to as telepresence, the feeling of being present at another location than the physical location of one's body; Van Erp et al., 2006;Jansen and van Erp, 2010).

To afford a flawless and seamless operation, a telerobotic system should be transparent (referring to an interface that appears to be imperceptible and almost non-existent to the operator), so that the user's performance is not influenced by the fact that the operation is mediated. In practice, there are conflicts between transparency and control, and compromises need to be made (Oh et al., 2018). Due to limitations of the human-machine interface, the communication channel and the robotic device, both the control signals and the multisensory (e.g., visual, auditory, haptic) feedback to the human operator may be delayed, out of sync, and of reduced quality and resolution compared to unmediated (direct) interaction (Niemeyer et al., 2016). This degraded interaction quality, in turn, degrades task performance (in terms of speed, precision, and accuracy) and increases the cognitive workload of the human operator.

Increasing the transparency of teleoperation systems is, therefore, an important research topic in engineering with a strong focus on technical solutions like increasing the bandwidth and reducing the latency of the communication. These solutions result in increased control of the operator and possibly enhanced performance. However, one could argue that ultimate transparency is accomplished when the operator does not even notice that the interaction is mediated and performed through a robot. In other words, optimal transparency implies that operators have the (illusory) experience that the robot's body and hands are their own body and hands. This is often referred to as the sense of embodiment. We use the term embodiment (the extent to which a physical or virtual representation in the real or mediated world is experienced as one's self; Aymerich-Franch et al., 2012) as an overarching construct including the senses of ownership, agency, and self-location (see Table 1 for the definitions of these constructs). The embodiment approach goes beyond technical solutions to increase transparency and includes recruiting brain mechanisms of telepresence (the perceived relation between one's self and the environment; Steuer, 1992) and body ownership (the perceived relation between one's self and one's bodily representation; Kilteni et al., 2012a). The classic Rubber Hand Illusion [RHI, in which an observer experiences body ownership of a rubber arm and hand when it is stroked simultaneously with the hidden own arm and hand: Botvinick and Cohen (1998)], and the correlation between embodiment of a prosthetic hand and task performance show the potential of an embodiment approach to increase transparency and performance in teleoperation. However, to the best of our knowledge, the direct link between embodiment and teleoperation performance has not been extensively investigated (or at all). We identify four premises that have to be met to come from the RHI to enhanced teleoperation performance. In this paper we focus on these premises and present relevant findings from the literature to support or disprove them (see Table 2).

**Table 1:** The different sensory experiences that are typically distinguished in embodiment research (after Kilteni et al., 2012a, see also Huang et al., 2017), their definition and interrelation, driving factors (origins) and associated measures.

**Primary experience**	**Components**	**Subcomponents**
**Sense of Embodiment (short: embodiment)** Definition: the feeling that a body is (part of) one's own biological body Origin: integration of sensory inputs	**Sense of self-location (short: self-location)** Definition: the feeling of location in space Origin: collocation between fake (virtual) and real body	**Sense of body location** Definition: the feeling where one's body is located in space Origin: temporal match between visual and tactile stimulation Measures: questionnaire items related to body location (Ehrsson et al., 2007); response to body location estimation tasks (Lenggenhager et al., 2007, 2009); (physiological) response to threat toward perceived body location (Ehrsson et al., 2007)
		**Sense of perspective location** Definition: the feeling where one's visual perspective is located in space Origin: visual feedback via 1PP or 3PP Measures: questionnaire items related to perceived viewpoint location (Galvan Debarba et al., 2017; Gorisse et al., 2017; Huang et al., 2017; Verhulst et al., 2018); rating perceived viewing direction (Pfeiffer et al., 2014)
	**Sense of ownership (short: ownership)** Definition: the feeling that non-bodily objects are part of one's own body Origin: realism of fake body and temporal match between visual and tactile stimulation Measures: questionnaire items gauging the extent to which fake body parts feel like one's own (Botvinick and Cohen, 1998; Longo et al., 2008; Aspell et al., 2009; Slater et al., 2010); proprioceptive estimations (Botvinick et al., 2005; Tsakiris and Haggard, 2005; IJsselsteijn et al., 2006); estimation of body parts' size (Normand et al., 2011); physiological responses to threat (Armel and Ramachandran, 2003; Petkova and Ehrsson, 2008; Slater et al., 2010; Yuan and Steed, 2010; Petkova et al., 2011a); changes in physiological signals (Moseley et al., 2008; Hohwy and Paton, 2010)	
	**Sense of Agency (short: agency)** Definition: feeling of being the author of an observed action Origin:efficiency of (motor) control Measures: questionnaire items related to motor control (Longo et al., 2008; Kalckert and Ehrsson, 2012).	

**Table 2:** The premises underlying the postulate that embodiment and teleoperation performance are directly linked, together with references providing supporting evidence.

**Premise**	**Supporting evidence**
1. The body representation is malleable and can include non-bodily objects	The body representation is malleable: e.g., (Lackner, 1988; Ramachandran and Hirstein, 1998; de Vignemont et al., 2005; Ehrsson et al., 2005b; Longo et al., 2009a) Non-bodily objects can be embodied: e.g., (Botvinick and Cohen, 1998; Slater et al., 2008; Petkova et al., 2011a; Nishio et al., 2012, 2018; Ogawa et al., 2012; Guterstam et al., 2013, 2015; Caspar et al., 2015; Kilteni et al., 2015; Ma and Hommel, 2015a; van der Hoort and Ehrsson, 2016; Aymerich-Franch et al., 2017a; Jazbec et al., 2017; Liepelt et al., 2017; Weser et al., 2017; Kondo et al., 2018a)
2. Embodiment can be evoked by mediated sensorimotor interaction	Embodiment can be evoked by mediated: - visuotactile stimulation: e.g., D'Alonzo and Cipriani (2012); (Petkova and Ehrsson, 2008; Slater et al., 2008; Kilteni et al., 2012a; Maselli and Slater, 2013; Keizer et al., 2016; Krom et al., 2019) - visuomotor and proprioceptive cues: e.g., (Tsakiris et al., 2006; Dummer et al., 2009; Sanchez-Vives et al., 2010; Slater et al., 2010; Petkova et al., 2011a; Walsh et al., 2011; Kilteni et al., 2012b; Alimardani et al., 2013; Ma and Hommel, 2013; Maselli and Slater, 2013; Steptoe et al., 2013; Kokkinara and Slater, 2014; Romano et al., 2015) - Visual perspective: e.g., (Lenggenhager et al., 2007, 2009; Petkova and Ehrsson, 2008; Slater et al., 2008, 2010; Aspell et al., 2009; Sanchez-Vives et al., 2010; Petkova et al., 2011a; Kilteni et al., 2012b; Ogawa et al., 2012; Maselli and Slater, 2013, 2014; Pomés and Slater, 2013; Piryankova et al., 2014; Liang et al., 2015; Pozeg et al., 2015; Pavone et al., 2016; Galvan Debarba et al., 2017; Gorisse et al., 2017, 2019; Chen et al., 2018) - Visual realism: e.g., (Farnè et al., 2000; Austen et al., 2004; Holmes et al., 2006; Haans et al., 2008; Petkova and Ehrsson, 2008; Longo et al., 2009b; Slater et al., 2009, 2010; González-Franco et al., 2010; Normand et al., 2011; van der Hoort et al., 2011; Farmer et al., 2012; Kilteni et al., 2012b, 2013; Banakou et al., 2013; Guterstam et al., 2013, 2015; Maister et al., 2013; Martini et al., 2013, 2015; Peck et al., 2013; Osumi et al., 2014; Lugrin et al., 2015; Argelaguet et al., 2016; Lin and Jörg, 2016; Bovet et al., 2018; Jung et al., 2018; Verhulst et al., 2018; Waltemate et al., 2018)
3. Once induced, embodiment is robust against inconsistencies between the robotic system and the operator's body	Embodiment is robust against inconsistencies in: - appearance: e.g., (Armel and Ramachandran, 2003; Hohwy and Paton, 2010) - location: e.g., (Armel and Ramachandran, 2003) - timing: e.g., (Maselli et al., 2016)
4. Embodiment enhances dexterous task performance	Embodiment enhances: - motor performance with a virtual limb (Grechuta et al., 2017) - prosthesis movement control: e.g., (Tan et al., 2014) - prosthetic object discrimination and manipulation: e.g., (Schiefer et al., 2016) - prosthetic manual accuracy and sensitivity: e.g., (Valle et al., 2018)

### Four Premises for Enhanced Teleoperation Through Embodiment

We postulate that embodiment of a remote manipulator can improve dexterous performance. Our reasoning goes along the following four premises (see Table 2):

1. The representation of the body in the brain is malleable and can include non-bodily objects like robotic hands and end effectors.
2. Embodiment can be elicited through mediated sensorimotor interaction (e.g., through indirect viewing and haptic displays).
3. Once established, embodiment reduces the operator's susceptibility to inconsistencies in size, movement, degrees of freedom, etc. of the teleoperated device. This may be considered an increase in transparency, even for teleoperated devices that do not resemble one's own arms and hands.
4. The strength and robustness of embodiment correlate positively with dexterous task performance.

In this paper, we review a wide variety of studies on embodiment, and we argue that each of our four premises is substantiated by experimental evidence (Table 2). In section The Sense of Embodiment, we focus on the different subcomponents of embodiment, the different measures of embodiment and theoretical frameworks to explain embodiment, including the predictive coding theory (Friston et al., 2011; Friston, 2012; Hohwy, 2013) as a useful framework to interpret and discuss the findings in embodiment experiments. Sections Premise 1: The Body Representation Is Malleable and Can Include Non-bodily Objects, Premise 2: Embodiment Can Be Evoked by Mediated Sensorimotor Interaction, Premise 3: Once Established, Embodiment Is Robust Against Inconsistencies, and Premise 4: Embodiment Enhances Dexterous Task Performance focus on the four premises and discuss the literature to substantiate or disprove them. In section Recent Developments we identify some relevant developments, and in section Discussion and Research Questions we formulate guidelines and research topics. In the final section Conclusions we present the conclusions of this study.

Summarizing, the main contribution of this paper are (i) the postulate that embodiment and teleoperation performance are directly linked, together with (ii) a first set of guidelines to apply embodiment in telerobotics. In addition, we identify some important research topics.

## The Sense of Embodiment

The sense of embodiment, or embodiment for short, has been defined as the sense that emerges when an object's properties are processed as if they were the properties of one's own biological body (Kilteni et al., 2012a). Combinations of sensory input from vision, touch, motor control, and proprioception are some of the mechanisms that are relevant to embodiment (for a review see Ehrsson, 2012). When the normal correlation between several sensory input streams is changed, the brain can re-evaluate the probabilities of input signals and create the illusion of embodiment of an artificial object (e.g., Kilteni et al., 2012a). Embodiment consists of three subcomponents (Kilteni et al., 2012a; see Table 1): sense of self-location (the subjective feeling where I am in space), sense of ownership (the illusory perception that non-bodily objects are part of my own body and are the source of associated bodily sensations), and sense of agency (the subjective feeling that I am the author of an observed action). The sense of self-location is in turn composed of two subcomponents (Huang et al., 2017; see Table 1): the sense of body location (the subjective feeling where my body is in space) and the sense of perspective location (the subjective feeling where my visual perspective is located in space).

### Measures of Embodiment

In the literature a range of different measures has been used to quantify embodiment, including subjective measures (i.e., self-report questionnaires; Longo et al., 2008; Petkova and Ehrsson, 2008; Slater et al., 2010; Normand et al., 2011), proxies, and behavioral and physiological responses (e.g., Ehrsson, 2007; Lenggenhager et al., 2007; Petkova and Ehrsson, 2008; Kilteni et al., 2012b; Tieri et al., 2017). Proxies include thresholds for temporal asynchrony detection between seen and felt self-generated movements (Hoover and Harris, 2016), perceived spatial (proprioceptive) “drift” toward the fake body or its parts (Botvinick and Cohen, 1998; Lenggenhager et al., 2007; Kilteni et al., 2012b), and drift in the perceived size of body parts (e.g., Normand et al., 2011; Kilteni et al., 2012b). Behavioral and physiological measures are for instance skin conductance (e.g., Armel and Ramachandran, 2003; Petkova and Ehrsson, 2008; van der Hoort et al., 2011; Honma et al., 2014; Riemer et al., 2015; Galvan Debarba et al., 2017; Grechuta et al., 2017), startle response (Riemer et al., 2015) and heart-rate deceleration in response to threat (Slater et al., 2010), changes in reaction times in visuotactile cross-modal congruency task (e.g., Pavani et al., 2000) and processing time of tactile stimuli (e.g., Moseley et al., 2008), time of onset of the illusion (e.g., Ehrsson et al., 2004; Yeh et al., 2017), rate of self-recognition (e.g., Tsakiris, 2008), local histamine reactivity (e.g., Barnsley et al., 2011), skin temperature reactivity (e.g., Moseley et al., 2008; Hohwy and Paton, 2010; Tieri et al., 2017), and neural activity in different brain areas (e.g., Ehrsson et al., 2004, 2007; Tsakiris et al., 2007). It has been suggested that most physiological measures reflect an illusionary disowning of the real hand in favor of the artificial substitute (Longo et al., 2008; Lane et al., 2017).

Although all the measures presented above are considered to reflect embodiment, they are not always clearly correlated and may not reflect a causal relation, as we will also show in the next section. This underlines that embodiment is a multidimensional construct and that a specific measure may only reflect part of the construct. The field can benefit from a consistent use of measures and a clear explanation of which aspects of embodiment they actually measure. A first step may be to link each measure to the components presented in Table 1. In the next section, we will further discuss the dissociations between the subcomponents of embodiment.

### Dissociations Between Subcomponents of Embodiment

As reflected by the frequent absence of correlation between different measures for ownership, the subcomponents of the sensation are not necessarily correlated. Neuropsychological studies have provided evidence for a dissociation between at least two body representations: a *body schema* which is used for motor action, and a *body image* which is used to make perceptual judgments (Kammers et al., 2006). Proprioception dominates vision for the body schema, while vision dominates proprioception for the body image (Kammers et al., 2009). Perceptual judgments (e.g., proprioceptive drift, depending on the body image) are more susceptible to ownership than actions (depending on the body schema; Kammers et al., 2009). Since they are also relevant in a teleoperation setting, we give three examples of dissociations: between ownership and self-location, between self-location and perspective-location, and between agency and ownership.

#### Dissociations Between Ownership and Self-Location

Ownership and self-location can become dissociated. For instance, changing the visual perspective in immersive Virtual Reality (VR) from first-person perspective (1PP) to third-person perspective (3PP) differentially affects ownership and self-location during full-body illusions: ownership is only consistently experienced from a 1PP while changes in perceived self-location are also perceived in a 3PP (Maselli and Slater, 2014).

#### Dissociations Between Self-Location and Perspective-Location

Self-location has been mainly investigated through experimentally induced out-of-body experiences. Out-of-body experiences can be triggered by delivering synchronous visuotactile stimulation on one's real body while displaying the corresponding visual stimuli at the location of the occluded physical body seen from a perspective of a person located behind one's back (Ehrsson, 2007). During an out-of-body experience, the sense of body-location and the sense of perspective location can become dissociated when people feel that they perceive the world from a location outside their physical body (Lenggenhager et al., 2007).

#### Dissociations Between Agency, Ownership, and Proprioception

Agency and ownership typically coincide, but both can also be dissociated (e.g., Abdulkarim and Ehrsson, 2016; Shibuya et al., 2017; for a review see Braun et al., 2018). Ownership is mainly determined by the realism of the fake body part (Argelaguet et al., 2016) and the temporal match between the perceived visual and tactile stimulation (Tsakiris and Haggard, 2005; Tsakiris, 2010; Kalckert and Ehrsson, 2012), whereas agency primarily arises from the match between predicted (intended) movements and actual kinesthetic feedback (i.e., the perceived efficiency of control: Frith et al., 2000; Argelaguet et al., 2016; Braun et al., 2018). This becomes manifest in the virtual hand illusion (VHI), where ownership is mostly determined by delays (temporal mismatches between visual and tactile stimulation; Ismail and Shimada, 2016; Shibuya et al., 2018), while agency is determined both by delays and by movement variability (visual-motor mismatches; Shibuya et al., 2018). Functional Magnetic Resonance Imaging (fMRI) studies have shown that ownership and agency are linked to different brain areas. Ownership involves activity in midline cortical structures while agency corresponds to activity in premotor areas (pre-SMA and BA6: Tsakiris et al., 2010), supporting the different underlying mechanisms of agency and ownership. Agency (Caspar et al., 2015), proprioceptive drift (Pavani et al., 2000; Holmes et al., 2006; Haans et al., 2008; Holle et al., 2011; Rohde et al., 2011; Kilteni et al., 2012b; Maselli and Slater, 2014) and slowing down of tactile processing (Folegatti et al., 2009) also occur in the absence of ownership. The other way around, passive movements abolished agency but left ownership intact, while incongruent positioning of the fake hand diminished ownership but did not eliminate agency (Kalckert and Ehrsson, 2012).

Drift and ownership can be dissociated in the RHI (Riemer et al., 2015; Romano et al., 2015) and in full-body illusions (Maselli and Slater, 2014) too. Changing the sensed position of the hand either toward or away from the rubber hand does not influence the subjective strength of the RHI (Abdulkarim and Ehrsson, 2016). Synchrony of stimulation does not affect the proprioceptive drift but increases the sense of ownership (Tamè et al., 2018). The time scales of ownership and proprioceptive drift are different as well: the illusion of owning the hand can occur as early as 6 to 11 s after the onset of simultaneous stroking (Ehrsson et al., 2004; Lloyd, 2007), while the proprioceptive drift continues to increase after the illusion has begun, sometimes for several minutes (Tsakiris and Haggard, 2005; Tsakiris et al., 2007). Also, startle, and skin conductance responses are correlated with ownership but not with proprioceptive drift (Riemer et al., 2015). This suggests that the sense of ownership and proprioceptive drift are mediated by different neural mechanisms (Ehrsson et al., 2004; Blanke, 2012). It seems that proprioceptive drift is the result of visuo-proprioceptive integration (Erro et al., 2018), while the sense of ownership results from multisensory (i.e., visual, proprioceptive, and tactile) integration (Ehrsson, 2020). Proprioceptive drift is a more sensitive measure for agency than subjective measures (Liepelt et al., 2017). Hence, proprioceptive drift is probably only useful as an indirect measure of the RHI in well-controlled experimental conditions and in combination with other measures like vividness ratings (Armel and Ramachandran, 2003; Moseley et al., 2008) and subjective methods like questionnaires (Longo et al., 2008). As for the defensive behavioral and physiological responses to threats to an artificial body part, it is not clear whether these actually reflect ownership or merely a bodily resonance (i.e., the activation of similar bodily states in the self when perceiving others: Riemer et al., 2015), possibly mediated by a mirror neuron system (Keysers and Gazzola, 2009; Serino et al., 2009; Brozzoli et al., 2013).

### Body Representation, Internal Models, and the Predictive Encoding Framework

It has been suggested that embodiment is mediated through a Bayesian perceptual learning process (Armel and Ramachandran, 2003) in which signals from different modalities that co-occur with a high probability in near-personal space (Ehrsson et al., 2004; Makin et al., 2008) are integrated in multisensory brain areas (premotor cortex and posterior parietal cortex: Ehrsson et al., 2004, 2005a). In this view, a body ownership illusion is simply the best explanation of new evidence from sensory input given an existing framework of the human body form (based on prior knowledge; Apps and Tsakiris, 2014; Samad et al., 2015; see also Riva, 2018). After the onset of the illusion, further incoming evidence is then incorporated into this false bodily self-representation and may override prior knowledge about the body model and its associated causal relations (Hohwy and Paton, 2010). However, even though the illusion can be very convincing, participants remain rationally aware that their body has not actually changed.

The increasingly popular neuroscientific theory of predictive encoding (Friston and Kiebel, 2009; Friston, 2012; Hohwy, 2013) is a useful framework to interpret and discuss data on embodiment experiments and to make predictions for embodiment effects in telerobotics. Contrary to the Bayesian perceptual learning paradigm, predictive encoding postulates that the brain generates models at each level of perceptual and cognitive processing to predict what information it should be receiving from the level below it (i.e., top-down). The brain then compares the actual bottom-up sensory information with the model predictions. Only discrepancies between both (referred to as prediction errors or surprises) are passed to higher levels where they are used to update the model or where they are “explained away” by activating a different model (e.g., “*this is not an external robotic end-effector but a hand that is part of my body”*). Model activation and updates are both directed at minimizing or suppressing prediction errors at a lower level (Friston and Kiebel, 2009; Friston et al., 2011). This generic theory can also be applied to embodiment in the sense that the brain effectively regulates and controls the body by actively maintaining an internal model (multisensory simulation or “body matrix”: Moseley et al., 2012) of the body and the space around it and by using this model to generate predictions of future sensory events. The body model is continuously updated in a bottom-up way by minimizing prediction errors that signal mismatches between its top-down predictions and actual sensory events (Apps and Tsakiris, 2014).

Embodiment of tools or virtual body parts appears to reflect the integration of two types of information: prior information that is innate or gained from experience (e.g., the appearance of our own body), and current multisensory information (Tsakiris, 2010). In this view multisensory stimulation is required to induce embodiment in a bottom-up way, while the preexisting body representation (self-image) modulates the illusion in a top-down way by limiting the extent to which foreign objects can effectively be integrated (Tsakiris and Haggard, 2005). Top-down mechanisms appear to constrain the integration process (IJsselsteijn et al., 2006), such that realistic (real and virtual) body parts are more easily attributed to the self than arbitrary objects (Armel and Ramachandran, 2003; Tsakiris and Haggard, 2005; Haans et al., 2008). In other words: small prediction errors can change the body model (resulting in embodiment of these objects), while large prediction errors are more difficult to explain away by the brain or at all and may prohibit embodiment. In the latter case, error signals will result in maintaining the a priori model (this is a robotic end-effector), and not in the activation of an embodiment model.

The advantage of adopting the predictive encoding theory as a framework for embodiment is that it unifies perception and motor control, which are also critical in telerobotics. The brain uses both to minimize prediction errors: perception to adjust the internal model and motor control to adapt to the environment or to fill in missing data. Both experimental and clinical research has shown that the neural substrates underlying embodiment significantly overlap with those driving fine motor control. fMRI and Positron Emission Tomography studies investigating the neural correlates of sensory integration driving body ownership, demonstrate that the RHI correlates with activity in the bilateral premotor cortex, the intraparietal sulcus, the sensorimotor cortex, the temporo-parietal junction and in the right posterior insula (Ehrsson et al., 2004, 2005a; Tsakiris et al., 2007, 2008). For the virtual hand illusion, the number of EMG activity onsets correlated positively with reported subjective strength of the illusion while the virtual arm was rotating (Slater et al., 2008). These findings suggest that ownership of virtual limbs and bodies engage the same perceptual, emotional, and motor processes that make us feel that we own our biological bodies.

## Premise 1: the Body Representation is Malleable and Can Include Non-Bodily Objects

Since we experience our body through a complex interaction of various perceptual streams including vision, touch, proprioception, and vestibular sensations, neither its perceived size nor its morphology are as rigid as we usually take for granted. By systematically manipulating the delivery of sensory stimuli in controlled experimental conditions, different illusions can be induced that significantly alter our bodily perception and representation.

### Malleability of the Body Representation

The apparent shape and orientation of the body can be changed by using muscle vibration to generate proprioceptive misinformation about limb position. For instance, vibrotactile stimulation of the biceps or triceps while the hand touches another non-moving body part creates the impression that the hand is extending away or toward the body, with the associated illusion that the touched body part is changing in size (Lackner, 1988; de Vignemont et al., 2005; Ehrsson et al., 2005b). Also, simultaneous vibration on the biceps and triceps muscle tendons can induce the perception of a shrunken arm (Longo et al., 2009a). A well-known example of changed sizes in body parts is the illusion of the long nose. This illusion occurs when a finger of a blindfold subject (S0) is manipulated by the experimenter to tap the nose of another subject (S1) who is sitting in front facing away from S0 while the experimenter simultaneously taps the nose of S0 (Ramachandran and Hirstein, 1998).

### Embodiment of Non-bodily Objects

Numerous studies have shown that is possible to include extracorporeal objects in our body representation. Ownership has been reported for objects such as fake limbs and robotic hands and arms, mannequins and virtual bodies and even empty volumes of space and invisible bodies (e.g., Caspar et al., 2015; Guterstam et al., 2015; van der Hoort and Ehrsson, 2016; Kondo et al., 2018a) through visuotactile stimulation or visuomotor synchronicity (see Kilteni et al., 2015 for a review of some illusions). This line of research started to expand significantly with the experiments of Botvinick and Cohen (1998) who found that watching a rubber hand being stroked in synchrony with one's own unseen hand can cause the rubber hand to be attributed to one's own body. Since then, this RHI or body ownership illusion (in short: ownership, see Table 1) has successfully been elicited for a virtual arm (Slater et al., 2008), robot hands (Guterstam et al., 2013) and robot arms (Aymerich-Franch et al., 2017a), whole mannequins (Petkova et al., 2011a), android robots (Nishio et al., 2012, 2018; Ogawa et al., 2012; Jazbec et al., 2017), virtual balloons and virtual squares (Ma and Hommel, 2015a), smartphones (Liepelt et al., 2017) and chopsticks (Weser et al., 2017). Hence, it appears that humans can incorporate a wide range of external objects into their body representation.

The extent to which non-human artifacts can be incorporated as a phenomenal extension of the self is clearly relevant to the area of telepresence (IJsselsteijn, 2005). Understanding the conditions under which integration can occur should guide the design of virtual environments, teleoperation, and mixed reality systems, and ways in which the body may be optimally represented in such mediated environments. In the remainder of this section we discuss the literature on ownership over non-bodily objects such as non-human-like avatars, robots, external, and additional tools.

#### Ownership Over Non-human Like Avatars

In a teleoperation setting it is relevant how non-human like an object can be (e.g., a robotic tool) and still evoke embodiment. A fake body (part) does not necessarily need to look realistic to evoke embodiment. Specific types of synchronous multisensory and sensorimotor stimulation can induce ownership over body parts or entire bodies with a shape, size, and symmetry that are different from one's normal body form (Longo et al., 2009b; González-Franco et al., 2010; Sanchez-Vives et al., 2010; Slater et al., 2010; Yuan and Steed, 2010; Normand et al., 2011; van der Hoort et al., 2011; Kilteni et al., 2012b; Piryankova et al., 2014; Verhulst et al., 2018). Several studies have also shown that we can incorporate more “limbs” than just prescribed by our body's morphology (e.g., Ehrsson, 2009; Newport et al., 2010; Guterstam et al., 2011; Folegatti et al., 2012; Chen et al., 2018). For instance, participants moving in synchrony with a humanoid avatar that featured a distinct and flexible tail-like appendage protruding from its coccyx, experienced a tail that they could control accurately and synchronously through hip movement (Steptoe et al., 2013). Changes over time are permissible as well: in a setup that combined VR with vibrotactile feedback, the illusion of an elongated arm was effectively induced by letting the participants concentrate on a virtual arm that was slowly elongated while a virtual ball was bouncing on the virtual hand (Ariza et al., 2016). Although similarity of the virtual avatar enhances the sense of embodiment in VR (Maselli and Slater, 2013), even bodies with extra limbs (Schaefer et al., 2009; Won et al., 2015), a tail (Steptoe et al., 2013), dragon wings (Egeberg et al., 2016), and animal bodies such as cows, spiders and bats (Ahn et al., 2016; Krekhov et al., 2019) can be embodied by participants.

#### Ownership Over Robots

Humans can identify with realistic androids (Nishio et al., 2012) as well as with non-human looking humanoid robots (Aymerich-Franch et al., 2015, 2017a). In the domain of robotics, embodiment has been induced toward teleoperated android arms or robot arms, either through visuotactile synchrony (Aymerich-Franch et al., 2017a), visuo-movement synchrony (Hellman et al., 2015; Romano et al., 2015; Aymerich-Franch et al., 2017a), or through a brain-computer interface (Alimardani et al., 2013). An ownership illusion was successfully induced not only for arms with a high resemblance to human arms in terms of shape (i.e., a hand with five fingers: Romano et al., 2015) and texture (Hohwy and Paton, 2010; Hellman et al., 2015), but also for non-human looking arms (Aymerich-Franch et al., 2017a). Presenting an arm that is attached to a robot in first-person perspective from the viewpoint of the humanoid robot probably contributes to its conceptualization as an “arm” and thus the development of functions associated to this limb, which in turn contributes to its integration as part of the body (Aymerich-Franch and Ganesh, 2016). A sense of full-body ownership and agency was induced for teleoperated life-size androids moving in synchrony with the participant's movements (Nishio et al., 2012; Jazbec et al., 2017). A first-person view with congruent visuo-auditory feedback effectively induced a sense of embodiment toward a moving humanoid robot (Aymerich-Franch et al., 2015). Participants experienced this sense of embodiment even when they did not control the robot. Furthermore, the sense of agency and body ownership were not affected by partial and delayed control of the robot (with delays of 0.5–2 s). In contrast, Arata et al. (2014) found that a robotic RHI significantly decreased at time delays over 100 ms.

Once it has been embodied, users can even experience haptic sensations from a non-anthropomorphic embodied limb or agent with only visual (and no haptic) feedback. For instance, participants reported haptic sensations in their real hand when they observed their robot avatar touching a curtain with its hand (Aymerich-Franch et al., 2017b). The ability to feel embodiment and ownership over non-human looking humanoids is important, since this type of robots may be more appropriate to perform certain functions than highly human-like ones (Złotowski et al., 2015).

#### Tools and Embodiment

Studies on tool use have shown that arbitrary mappings between motor intentions and sensory events can be learned (Sato and Yasuda, 2005; Sato, 2009; Spengler et al., 2009) and thereafter predicted, and novel internal models can be acquired to facilitate tool use (Imamizu et al., 2000; Wolpert et al., 2011). These findings are summarized by the functional body model hypothesis (Aymerich-Franch and Ganesh, 2016), which states that perceived entities with functional properties that match those of our own body parts can be *embodied* by our brain, while tools that afford different functionalities can *modify* our internal body representation (body model) leading to perceptual changes that depend on the shape and functionality of the tools. It appears that arbitrary tools can be embodied provided that the actions they afford match the task at hand (Berti and Frassinetti, 2000; Cardinali et al., 2012).

Tool use can update the brain's representation of the body's morphology and kinematics such that the modified representation reflects an incorporation of the tool into the body representation. As a result, tool use can extend peripersonal space to include the space reachable by the tool (Berti and Frassinetti, 2000). This extended body model can, in turn, be recalibrated through manipulation of the multisensory stimuli perceived through a tool: the RHI was for instance successfully induced for participants looking at a rubber hand holding a pair of chopsticks while holding an identical pair in their own hands (Weser et al., 2017). Hence, it appears that the brain treats the representation of an embodied tool in the same way as the representation of the (artificial) hand wielding it.

#### Prosthetics

Embodiment is of particular interest for prosthetics, where one of the main goals is to restore the motor and sensory functions of a lost limb with an artificial substitute that feels and acts like the lost real one. Hence, the artificial body part should become integrated into the internal representation of the body, through a modification of its sensorimotor cortical representation. Embodiment has successfully been achieved in transradial amputees by stroking specific points on the residual limb (Ehrsson et al., 2008) and by using a prosthesis equipped with artificial sensors that provide synchronized vibrotactile feedback on the stump (D'Alonzo and Cipriani, 2012). Using a bidirectional neural-machine interface, it has recently been shown that amputees intuitively acquire a sense of agency and improved functional motor control over robotic hands when kinesthetic feedback is provided in the form of vibration-induced perceptual movement illusions (Marasco et al., 2018).

Myoelectric prostheses record, process and decode electrical activity from the user's muscles to control the device's actions. Active prosthetics close the prosthesis control loop by also providing sensory feedback to the user. Myoelectric control over a robotic arm induced a significant sense of embodiment in both able-bodied participants and amputees, as manifested by high degrees of ownership and agency (Sato et al., 2018).

### Conclusion on Premise 1

The above studies show ample support that the representation of the body is indeed malleable and may also include non-bodily objects like tools and prosthetic limbs. Objects that do not resemble body parts can be included, provided that ownership has been induced beforehand (Hohwy and Paton, 2010). This suggests that different substrates mediate the initial elicitation of the BOI (Ehrsson et al., 2004) and its maintenance, once established (Tsakiris et al., 2006, 2007, 2008; Ehrsson, 2012). It appears that embodiment can be induced for any type of object (even virtual balloons or squares and smartphones: Ma and Hommel, 2015a; Liepelt et al., 2017; Weser et al., 2017) as long as one can control the relevant features and behaviors of that effector (i.e., as long as one has objective agency over the effector: Ma and Hommel, 2015b).

## Premise 2: Embodiment Can be Evoked by Mediated Sensorimotor Interaction

In section Body Representation, Internal Models and the Predictive Encoding Framework, we explained that according to the predictive encoding framework, bottom-up sensory cues can drive the activation of an embodiment model. In a telerobotics situation, these cues will be mediated or even virtual. In the previous chapter, we already presented several studies that used immersive VR, showing that cues can be indirect. In this chapter, we look more closely at the different cues in relation to the predictive encoding framework and the possible effect of mediation.

According to the predictive encoding framework, bottom-up sensory signals can enforce changes in the internal model and therewith mediate embodiment. A relevant question is how, how quickly, to what extent, and how robustly the different sensory signals evoke embodiment. Embodiment is typically generated by either visuotactile or visuomotor information, but visuo-proprioception and visual perspective can also have a significant impact. Unmediated visuotactile stimulation is the main sensory input for embodiment in studies related to the classic rubber-hand paradigm (e.g., Botvinick and Cohen, 1998). More recently, different paradigms have successfully been deployed to induce embodiment. In virtual reality, high precision visuomotor synchronization is now generally used to induce the sense of avatar embodiment (e.g., Kilteni et al., 2012a). It appears that the mere view of a rubber hand near the participant's own hand (e.g., Pavani et al., 2000; Holmes et al., 2006; Rohde et al., 2011) or the mere sight of a mannequin body from a 1PP perspective (e.g., Carey et al., 2019) can also be sufficient to induce a significant ownership illusion.

In this section we compare findings in the literature on the effect of different (combinations of) sensory inputs on embodiment, and discuss which sensory modalities are critical and which are sufficient to induce embodiment. More specifically, we look at the following questions: (a) how do different combinations of sensory cues contribute to embodiment, (b) is embodiment possible without a particular sensory cue, (c) what is the effect of inconsistencies in specific sensory cue combinations, and (d) are there differences between direct, unmediated cues, and mediated or virtual cues.

### Visuotactile Stimulation

Visuotactile stimulation typically involves synchronous stroking of the hand and its representation (e.g., rubber hand or virtual hand). Different tactile stimuli like stroking (e.g., Botvinick and Cohen, 1998), tapping (e.g., Haans et al., 2012) and even the presentation of painful tactile stimuli (pinpricks: Capelari et al., 2009) can all be used to induce the RHI. Affective touch (slow stroking) induces a stronger sense of ownership than non-affective touch (e.g., either fast stroking or tapping), both for the RHI (Haans et al., 2012; Crucianelli et al., 2013, 2018; Lloyd et al., 2013; van Stralen et al., 2014) and for a virtual full-body ownership illusion (de Jong et al., 2017). The RHI can also be induced through (dynamic or static) active self-touch realized by a robotic master-slave system (Hara et al., 2016).

The RHI depends on the congruency of the tool used to stroke the real and fake hands. The RHI is diminished when tools are used that are incongruent with respect to their visual appearance and predicted tactile stimulation (e.g., touching the dummy with a pencil and the real hand with a paintbrush) relative to when they are congruent (Ward et al., 2015). In a study by D'Alonzo and Cipriani (2012), synchronized vibrotactile feedback and visual stimulation effectively induced the RHI despite sensory substitution and the modality-mismatched nature of the feedback to the real hand. The RHI can also be induced using somatic signals only, that is without visual cues, by moving a blindfolded participant's index finger so that it touches a rubber hand while the experimenter simultaneously touches the participant's real other hand (Ehrsson et al., 2005a).

But is visuotactile synchrony actually required? In some studies, spatiotemporal mismatches between vision and touch significantly inhibit the RHI (Armel and Ramachandran, 2003; Ehrsson et al., 2004; Slater et al., 2008). Similar results were found for full ownership toward plastic mannequins seen from a 1PP: participants perceived the mannequin's body as their own body when the real and the fake abdomen part were touched synchronously, but not when they were touched asynchronously (Petkova and Ehrsson, 2008). However, other studies show that although synchronized multisensory cues (visuotactile or visuomotor) can strengthen the illusion, they are not required for ownership to occur (Maselli and Slater, 2013; Keizer et al., 2016). When the fake body (part) is realistic and overlaps in space with the real body counterpart, ownership can even be induced in presence of asynchronous visuotactile stimulation (Maselli and Slater, 2013; Krom et al., 2019).

In conclusion: while visuotactile synchrony can significantly enhance the illusion, it is not crucial to experience embodiment, and embodiment may even occur with asynchronous visuotactile cues in the presence of additional congruent cues. There are no apparent differences between direct and mediated or virtual cues.

### Visuomotor and Proprioceptive Cues

Visuomotor correlations are extremely efficient in eliciting full body ownership (Slater et al., 2010; Kokkinara and Slater, 2014), thanks to the rich information processing involved in the sensorimotor control loop. A feeling of embodiment can be induced for a wide variety of virtual objects if the perceived actions of the object are predictable in accordance with the intentions of the individual (Short and Ward, 2009). More specifically, a correlation between efferent (operator's intended motion) and visual afferent (visual perception of actual motion) information is sufficient to trigger the illusion of body ownership (Alimardani et al., 2013, 2015). As a result, ownership can be elicited by congruent visuomotor cues alone (through a synchronized motion of the real and the fake body parts), with no need for further sensory cues (Tsakiris et al., 2006; Dummer et al., 2009; Walsh et al., 2011; Maselli and Slater, 2013). Synchronized movements of real and fake body parts (e.g., by tracking the movements of the real ones to steer the fake ones) can induce embodiment in the absence of visuotactile stimulation (e.g., Tsakiris et al., 2006; Dummer et al., 2009; Sanchez-Vives et al., 2010; Ma and Hommel, 2013; Romano et al., 2015). In this case, the visual feedback corresponds to the perceived movements of the fake body parts. For instance, when operators watch a teleoperated robot copying their own movements, the match between the motion commands (efferent predictive signals) and the sensory feedback from the motion (visual afference from the robot's body and proprioceptive afference from the operator's own body) induces a sense of agency over the robot's actions and ultimately results in a sense of embodiment of the robot's body (Alimardani et al., 2013). Also, synchronized visual and non-cutaneous proprioceptive cues can induce embodiment: congruent movements performed under digital nerve block induced ownership for an artificial finger that was even stronger than the ownership that was induced by congruent movements performed with an intact finger (Walsh et al., 2011).

Using a virtual hand-arm model for visual stimulation in combination with vibrotactile feedback through a data-glove, Padilla et al. (2010) showed that synchronized movements in combination with self-inflicted tactile stimulation (through touching virtual objects) effectively induced a significant ownership. Visuomotor synchrony effectively induced ownership over an extended-humanoid avatar in immersive VR (Steptoe et al., 2013). In addition, visuomotor synchrony between movements of real and virtual hands induced illusions of ownership, proprioceptive drift and agency of virtually presented hands (Sanchez-Vives et al., 2010; Rognini et al., 2013). A similar study found that visuomotor congruency between movements of the real hand and a detached myoelectric-controlled robotic hand induced proprioceptive drift but not ownership, whereas the illusion did not occur for incongruent movements (Romano et al., 2015). The difference between these results may be caused by differences in the degree of realism of the fake hand: while the virtual hands had a realistic appearance (Sanchez-Vives et al., 2010; Rognini et al., 2013), the robotic hand showed mechanical and electronic components (Romano et al., 2015).

Concerning proprioception and visuo-proprioceptive congruency, ownership has shown to be relatively insensitive for small discrepancies in position (Ehrsson et al., 2004; Rohde et al., 2011; Maselli and Slater, 2014) and orientation (Petkova et al., 2011b; Brozzoli et al., 2012; Ide, 2013; Ionta et al., 2013; Blom et al., 2014) between the real and fake body parts, but decreases in strength for larger spatial discrepancies (Lloyd, 2007; Bergström et al., 2016).

The distribution of embodiment in the RHI across stimulated and non-stimulated fingers depends on the kind of stimulation. Both touch and passive movement produce a relatively fragmented perception of one's own body, in which a sense of ownership is associated with individual body parts and does not transfer to other parts. However, active movement can integrate distinct body parts into a coherent, unified awareness of the body (Tsakiris et al., 2006): tactile and passive stimulation-induced localized proprioceptive drifts, specific to the stimulated digit, while active movement of a single digit spread the proprioceptive drift across the whole hand. This kind of fragmented perception can be a limitation of the embodiment in teleoperation applications when different body parts are receiving different levels (e.g., intensity, realism, congruency) of (artificial) feedback.

In conclusion, the above results show that visuomotor and proprioceptive synchrony significantly enhance embodiment and are sufficient—but not required—cues. It is not yet clear whether inconsistencies in these cues can prohibit embodiment from occurring. Mediated cues seem to have the same effect as direct cues.

### Visual Perspective

The perspective from which the body(-part) is seen is another important modulator of illusory body ownership. Ownership of a virtual body can be achieved in both 1PP (Petkova and Ehrsson, 2008; Slater et al., 2010; Petkova et al., 2011a; Maselli and Slater, 2013) and in 3PP (Lenggenhager et al., 2007, 2009; Pomés and Slater, 2013; Gorisse et al., 2017, 2019). Participants can also experience body ownership over a teleoperated android robot in both 1PP and in 3PP (Ogawa et al., 2012). However, ownership is typically stronger from a 1PP compared to a 3PP (Petkova and Ehrsson, 2008; Slater et al., 2010; Petkova et al., 2011a; Pozeg et al., 2015), while a 1PP and a 3PP induce the same sense of agency and spatial presence (Gorisse et al., 2017). A 1PP (compared to a 3PP) generally yields a stronger embodiment in terms of self-location and ownership (Galvan Debarba et al., 2017) and enables more accurate interactions due to a better perception of the arms and the hands of the users' avatars (Gorisse et al., 2017), while a 3PP provides a better awareness of the environment. In essence, 1PP can be considered as congruent visual perspective and 3PP as incongruent. On a side note, the vast majority of studies in this area used avatars and immersive virtual reality, showing that the sensory cues can be mediated.

Several studies suggest that in an immersive VR environment a 1PP is already sufficient for embodying an avatar with human features (Maselli and Slater, 2013; Piryankova et al., 2014). A 1PP over a fake humanoid body induces even higher levels of experienced ownership, compared to a 3PP (Slater et al., 2010; Petkova et al., 2011a; Maselli and Slater, 2014; Pavone et al., 2016). Some degree of consistency between the fake body part and the human's own is required to induce embodiment: if the discrepancy (i.e., the prediction error in terms of the predictive encoding framework) is too large, embodiment is not elicited. Ownership is less likely to occur when the apparent visual location or orientation of a body part conflicts with its veridical location (Pavani et al., 2000). In a 1PP, ownership can be induced over a virtual arm that moves with the same spatiotemporal pattern as the real one independent of the degree of congruency of the visuotactile feedback (Slater et al., 2008; Sanchez-Vives et al., 2010; Kilteni et al., 2012b). A 1PP (i.e., an avatar being spatially coincident with the position of the participant) of a realistic virtual body substituting the participants' own body induces an illusory feeling of ownership and changes in body representations (Serino et al., 2016), even after asynchronous stimulation (Slater et al., 2010; Maselli and Slater, 2013; Serino et al., 2016).

Using a system that captures human body movement and generates an immersive 1PP view of one's body position with a small (forward or backward) shift in time, it was found that spatiotemporal deformations of the body representation induced distinct physical sensations (Kasahara et al., 2017). Viewing a prediction of one's body image in the future induced lighter, nimble and vitalized sensations, whereas past views evoked heavier, dull, and diminished sensations.

Embodiment can occur with respect to a distant body, seen from a 3PP, when additional reinforcement in the form of synchronous visuotactile information is provided (Lenggenhager et al., 2007, 2009; Aspell et al., 2009) or when the virtual body preserves spatial overlap with the physical body (Maselli and Slater, 2014). The latter indicates that 3PP alone is not enough, and additional boundary conditions apply. Postural congruency does not appear to be essential (Liang et al., 2015; Chen et al., 2018). While ownership over a body seen from a 1PP and a 3PP are both supportable (Pomés and Slater, 2013), a 3PP of the virtual body sometimes appears to break the illusion (Petkova and Ehrsson, 2008; Slater et al., 2010; Petkova et al., 2011a).

While the sense of ownership and sense of self-localization are tightly coupled in normal conditions, they can become dissociated during virtual out-of-body experiences (e.g., Maselli and Slater, 2014). In an out-of-body experience, the perspective switches from 1PP to 3PP, making it possible to perceive the world from a location outside the own body and/or even see one's own body. Thus, 3PP alone is not sufficient to evoke embodiment and is not a strong cue either. 3PP may even break existing embodiment.

In conclusion on visual perspective, the above results show that a 1PP is a sufficient cue to evoke embodiment but is not strictly required, as embodiment also occurs in the absence of visual cues or with an incongruent visual perspective (i.e., 3PP). However, 3PP alone is not sufficient to evoke embodiment and is not a strong cue either. 3PP may even break existing embodiment. In this respect, mediated vision is as good as direct vision.

### Visual Realism (Similarity)

Although some degree of connectivity (Perez-Marcos et al., 2012) and anatomical correspondence between the real and the dummy body part must be respected for embodiment to occur (Tsakiris and Haggard, 2005), a growing body of evidence shows that the embodiment is highly flexible and does not require a high degree of visual realism for its induction (Slater et al., 2009). It is, for instance, possible to experience ownership over joke-shop rubber hands (Farnè et al., 2000), stuffed washing gloves (Austen et al., 2004), zombie hands and wooden blocks (Lin and Jörg, 2016), arms that appear much longer than normal (Kilteni et al., 2012b), that look injured or particularly hairy (Osumi et al., 2014), that have different (Holmes et al., 2006; Farmer et al., 2012) or dynamically changing (Martini et al., 2013) skin color or luminance (Longo et al., 2009b), that are semitransparent (Martini et al., 2015), or which are even completely invisible (Guterstam et al., 2013). Also, ownership can be experienced over virtual bodies of the same (Petkova and Ehrsson, 2008; González-Franco et al., 2010) or opposite (Slater et al., 2010) sex, over virtual bodies of a different race (Longo et al., 2009b; Farmer et al., 2012; Kilteni et al., 2013; Maister et al., 2013; Peck et al., 2013), different shape and size (Normand et al., 2011; van der Hoort et al., 2011; Banakou et al., 2013; Verhulst et al., 2018), invisible virtual bodies (Guterstam et al., 2015), of dolls and giants (van der Hoort et al., 2011), of robots and block-men (Lugrin et al., 2015).

A correct representation of self-contact appears to support a strong sense of embodying an avatar during immersion in VR (Bovet et al., 2018). Another study found a significant shape by texture interaction: a natural skin texture increased the strength of the RHI for a hand-shaped object, but not for a non-hand-shaped object (Haans et al., 2008). Using a high-fidelity personalized hand in augmented virtuality (a VE enhanced with real objects) resulted in significantly higher accuracy in object size estimation, in addition to higher degrees of spatial presence and body ownership (Jung et al., 2018). Also, personalized avatars significantly increased virtual body ownership (Waltemate et al., 2018). However, in several other RHI studies regarding realism, different skin colors and hand shapes of believable rubber human hands did not affect the strength of the RHI (Longo et al., 2009b; Farmer et al., 2012).

Since objective agency does not depend on similarity or realism, agency can even be larger for non-realistic body parts when these provide a better feeling of control (Argelaguet et al., 2016; Lin and Jörg, 2016). These observations agree with physiological properties of cells in premotor and intraparietal cortices which drive the fast localization of limbs in space (Graziano, 1999; Graziano et al., 2000), but are not involved in visual object recognition and the visual scene analysis. Moreover, it means that embodiment may be a valuable paradigm for applications requiring a high degree of agency, since agency is not restricted to realistic simulations, high definition visual displays or even anthropomorphic body parts, but can also be induced for artificial or (low resolution) virtual entities.

In conclusion: (visual) likeness helps to evoke embodiment but is not required, and cues may even be incongruent. Mediated vision is as good as direct vision.

### Comparison of Sensory Modalities

As mentioned above, factors known to contribute to embodiment include synchronous visuotactile information, visuomotor synchrony and visuo-proprioceptive match, visual perspective on the body, and visual appearance of the body. Data also show that modalities may have different effects on the strength and robustness of the effect and that none of them is strictly required for embodiment to occur. This gives rise to two important questions: is there a simple additive model of sensory cues and is the strongest and most robust embodiment achieved when all cues are present? And how do these factors quantitatively compare in evoking embodiment? The latter requires studying the main effects of the sensory modalities and their interactions in a single, elaborate experiment. Such an ultimate experiment has not been done yet. However, we can make a rough ordering of the modalities (from strong to weak, based on the findings above and discussed below): 1PP visual perspective, visuo-motor / proprioception synchrony, visual-tactile synchrony, and visual similarity.

Perspective (specifically 1PP) dominates movement and touch as an explanatory factor for ownership (Slater et al., 2010). As noted before, a 1PP over a realistic fake human body or body part overlapping the real one is sufficient to elicit ownership (Maselli and Slater, 2013). In that case, there is no need for an additional contribution of congruent visuotactile or sensorimotor cues. However, the contribution of congruent multisensory and/or sensorimotor cues is required when the level of realism of the virtual body is not high enough or when there is no (or insufficient) spatial overlap between the two bodies (Blom et al., 2014). Current evidence suggests that (in the context of 1PP) visuomotor congruence contributes significantly more to ownership than visuotactile synchrony does (Slater et al., 2010; Petkova et al., 2011a; Kilteni et al., 2012b; Kokkinara and Slater, 2014). Disruption of ownership by asynchronous visuotactile stimulation can be overridden by synchronized visuomotor stimulation (Kokkinara and Slater, 2014). The RHI illusion is stronger in an active movement condition compared to a passive condition, indicating that both efferent and afferent information (proprioception) contribute to body perception (Raz et al., 2008; Dummer et al., 2009). Haptic feedback (e.g., tactile and force feedback) during the active movement also contributes significantly to the feeling of ownership (Raz et al., 2008).

As mentioned above, tactile feedback is not a strict requirement—operators watching a teleoperated android robot (Nishio et al., 2012) or a virtual body (González-Franco et al., 2010; Verhulst et al., 2018) moving in synchrony with their own movements also experience embodiment. This still occurs when there are discrepancies in position and orientation between the fake and real body parts, although it is less strong in these conditions (Bergström et al., 2016). Synchronous visuotactile stimulation can overcome spatial mismatches and induce the illusion when the rubber hand is placed in an anatomically plausible posture but in a different position (Ehrsson et al., 2004; Rohde et al., 2011) or in a different orientation (Brozzoli et al., 2012; Ide, 2013; Ionta et al., 2013) from the real one. The RHI even occurs when the tactile stimulation is merely suggested (White et al., 2017; Smit et al., 2018).

An asynchronous multimodal stimulation is typically adopted as the control condition in which no sense of ownership is expected to arise since the error signal is too large to trigger the embodiment model. However, despite the large prediction error for an embodiment model, the asynchronous condition does not always result in a significantly lower sense of body ownership compared with the synchronous one (Hara et al., 2015; Kokkinara et al., 2015; Pozeg et al., 2015). This confirms the inference based on the predictive encoding framework that in principle no sensory signals are strictly required, and all sensory signals can potentially activate the embodiment model. However, some modalities may have a stronger (i.e., a larger weight) effect than others, as is common in multisensory integration in general and related to differences in signal noise.

### Conclusions on Sensory Modalities and Mediation of Cues

To conclude, embodiment can be induced and enhanced with different (combinations of) sensory input. In accordance with predictions based on the predictive encoding framework, not all sensory modalities are needed in all situations, and the information transferred by the different modalities appears to reflect different aspects of embodiment. The available data suggests (a) that the sensory information is not redundant, but complementary, (b) the input of the different modalities is weighted, and (c) there is some form of additive combination of cues. It is also evident that there are not yet large-scale experiments reported investigating all relevant factors and their interactions.

When we interact with virtual or remote environments using intuitive interaction devices, isomorphic to our sensorimotor abilities, a real-time, reliable, and persistent chain of user action and system feedback will effectively integrate the technology as a phenomenal extension of the self (IJsselsteijn et al., 2006). Hence, embodiment can also be established through artificial tactile stimulation (i.e., through a tactile display) or visuomotor interaction with indirect viewing, i.e., both the sensory-motor and visual input can be mediated. This suggests the possibility to induce and apply embodiment in teleoperation settings, where the operator typically receives mediated visual/motor/tactile feedback. The data presented in the sections above clearly substantiate Premise 2: embodiment can be evoked by mediated sensorimotor interaction.

## Premise 3: Once Established, Embodiment Is Robust Against Inconsistencies

There are limits to the discrepancies for which ownership can still be induced: the illusion does not arise for fake body parts that have an overly unrealistic (e.g., a wooden stick: Tsakiris and Haggard, 2005) or abstract (Yuan and Steed, 2010) appearance, that have impossible postures (Ehrsson et al., 2004; Tsakiris and Haggard, 2005; Costantini and Haggard, 2007) or that are located outside the peripersonal space (Lloyd, 2007). However, once a basic ownership has been induced, these restrictions no longer hold, and it is possible to extend ownership over non-body objects like a table surface (Armel and Ramachandran, 2003) or a box (Hohwy and Paton, 2010) and over objects positioned at anatomically impossible locations (e.g., outside the peripersonal space: Armel and Ramachandran, 2003). This indicates that once the embodiment model is activated, mismatching sensory cues will lead to adjustments of the model, as long as the mismatch between the predicted and actual sensory information is below a certain threshold. In other words: inconsistencies in size or appearance, for example, of an embodied object will not necessarily result in abandoning the embodiment model in favor of a “*this is an external object”* model. An interesting hypothesis would then be that a step-by-step introduction of a large inconsistency (e.g., slowly increasing of arm length (Ariza et al., 2016), will be incorporated in the embodiment model easier than the abrupt introduction of the same inconsistency.

While agency and ownership tolerate quite large temporal delays (up to 350 ms, Shimada et al., 2009; Ismail and Shimada, 2016), perceived simultaneity and motor performance both break down at much smaller delays (above 75 ms; Waltemate et al., 2016). Interestingly, illusory ownership also modulates the temporal constraints for multisensory integration: during the illusion, the temporal window for visuotactile integration of body-related cues expands (Maselli et al., 2016). As a result, once agency and ownership have been established, people fail to notice asynchronies in visuotactile stimulation that are otherwise (in the absence of the illusion) detected (Maselli and Slater, 2013). Note that this finding may be essential in teleoperation performance, since it may help to alleviate operator restrictions imposed by system limitations.

These examples show that premise 3 is supported by experimental evidence.

## Premise 4: Embodiment Enhances Dexterous Task Performance

Both experimental and clinical research have shown that the structures underlying body ownership significantly overlap with those mediating motor control, including the parietal and ventral premotor cortices, Temporal Parietal Junction (TPJ) and the insula (Ehrsson et al., 2004, 2005a; Tsakiris et al., 2007). This suggests that embodiment may influence motor performance. However, the relation between (degree of) embodiment and (dexterous) task performance has hardly been studied. Some recent studies indeed observed that the degree of ownership over a virtual limb directly modulates performance in a simple sensorimotor task: higher degrees of ownership resulted in faster reaction times (Grechuta et al., 2017). This implies that body ownership is not exclusively a perceptual and/or subjective multimodal state, but that it is tightly coupled to systems for decision-making and motor control.

Prostheses with implantable peripheral nerve interfaces that provide sensory feedback in the form of neural stimulation elicit somatotopic sensations similar to natural ones, affording intuitive control over bidirectional prosthesis. This fosters prosthesis embodiment (Schiefer et al., 2016; Rognini et al., 2019) and leads to improved prosthesis movement control (Tan et al., 2014) and improved object discrimination and manipulation (Schiefer et al., 2016). Furthermore, a recent study showed that an intraneurally controlled bidirectional hand prosthesis yielded both high manual accuracy and rich tactile sensitivity combined with an enhanced sense of embodiment (Valle et al., 2018).

These studies show that there is a link between embodiment and performance for prostheses. Although this concerns a limited number of studies that do not involve telerobotics, we consider them as indirect support for premise 4. However, definite proof must be obtained by investigating the link between embodiment and dexterous performance in a telerobotics situation directly.

## Recent Developments

In this section, we present recent developments that may become relevant for embodiment in telerobotics in the near future.

### Brain-Computer Interfaces

Brain-computer interfaces (BCIs) are systems that capture brain signals (either invasively or non-invasively) and translate them into commands for control of external devices (van Erp et al., 2012). Although the concept of direct brain control over artificial limbs or devices is much older, the integration with multisensory cues and including e.g., haptic feedback dates back to the beginning of this century (see van Erp and Brouwer, 2014 for an overview). BCI technology enables users to move prostheses (Kansaku et al., 2015), to navigate through virtual environments (Pfurtscheller et al., 2006; Leeb et al., 2007a,b; Lécuyer et al., 2008) and to control robotic devices in telepresence settings by mere thought (Escolano et al., 2012; Alimardani et al., 2013; Kishore et al., 2014). BCI control of either the movements of a virtual body (Perez-Marcos et al., 2009) or its navigation through a virtual environment (Ori et al., 2014) can effectively induce an experience of agency and embodiment.

Similar effects were found for humanoid robots. A study on an EEG-based BCI for the teleoperation of a humanoid robot found that the correlation between motor intentions (imagining a movement) and the visual feedback of the robot's motions (watching the robot perform the intended movement) induced a sense of body ownership (Alimardani et al., 2015). The illusion of embodiment was only significant when the robot's hands moved in agreement with the operator's intentions. The strength of the illusion correlated with the operator's BCI performance and sense of agency.

### Toward Superhuman Morphologies

Participants that simultaneously viewed the experimenter's hands from a 1PP and their own hands from a 3PP (using an HMD) while all hands tapped their index fingers, experienced a four-hand illusion (Chen et al., 2018). Participants also experienced high levels of ownership and agency over a six-digit virtual hand (Hoyet et al., 2016) and a third arm, both in reality (Schaefer et al., 2009) and in augmented reality (Rosa et al., 2019). In addition, Kishore et al. (2016) found that it is possible to concurrently experience full-body ownership over multiple artificial bodies (two different humanoid robots located in two distinct places and a virtual body represented in immersive VR). Together, these findings open the way for superhuman (robot) morphologies such as larger, smaller, stronger, more agile or flexible, more numerous or semi-transparent limbs.

### Augmented and Extended Reality (AR, XR)

In Chapter 4, we concluded that there are no differences between (1) direct or mediated sensorimotor interactions and between (2) real and virtual cues. It is likely that this also holds for Augmented Reality (AR; Rosa et al., 2015; Škola and Liarokapis, 2016), which opens up new opportunities in telerobotic applications. For instance, AR techniques can be used to manipulate the appearance and perceived tactile properties of artificial body parts or tools and therewith the strength of the ownership illusion in a similar vein as has been demonstrated with VR, and the combination of VR/AR and robotics affords automated induction of embodiment. For instance through robot arms or servo motors that touch both the real and the fake body parts (Tsakiris et al., 2008; Rohde et al., 2011), or through mechanical vibrators (vibrotactile actuators) attached to the real body (Pabon et al., 2010; Evans and Blanke, 2013; Maselli and Slater, 2013; Huisman et al., 2016). Automated vibrotactile stimulation can be very effective due to the high synchronicity that can be achieved between the automatic tactile stimulation and the displayed audio-visual events (Ariza et al., 2016). For instance, synchronized vibrotactile feedback through a data-glove effectively induced significant ownership for a virtual hand-arm model (Padilla et al., 2010). Slow stroking touch tuned to stimulate the C-tactile afferents can effectively be delivered by a robotic tactile stimulator (Pawling et al., 2017) or a vibrotactile sleeve (van Erp and Toet, 2015). As a result, automated stroking delivered by robotic hands (Rohde et al., 2013) or dedicated stroking devices (Salomon et al., 2013) can effectively induce the RHI.

### Behavioral Adjustment to Body Appearance

Full body ownership illusions can lead to substantial behavioral changes depending on the appearance of the virtual body. Participants who experienced ownership over a casually dressed dark-skinned body in an immersive VR setting showed a significant increase in variation and frequency of movement while playing hand drums, compared to participants who were embodied by a formal suited light-skinned body (Kilteni et al., 2013). Teleoperators of android robots tended to adjust their body movements to the movements of the robot (Nishio et al., 2012). For example, they talked slowly to synchronize with the geminoid lip motion and copied the robot's small movements. This suggests that the affordances of artificial bodies can be adapted to by operators, but they should be tuned to the task at hand so that they do not restrict the operator's task performance. Furthermore, the effects may not be symmetrical. For example, the perceived size of the fake body part appears to have an asymmetrical effect on the RHI: the illusion occurs when the fake arm is larger than one's own but not when it is smaller (Pavani and Zampini, 2007).

## Discussion and Research Questions

### Subcomponents of Embodiment and Task Performance

We postulated that embodiment can improve dexterous performance in teleoperation tasks, by making the interface transparent through the illusion that the tasks are performed with (a part of) one's own body. As shown in Table 1, the sense of embodiment consists of three components: the senses of ownership, agency, and self-location. We also showed that there is ample evidence that these three subcomponents are dissociated. Dependent on the task and the nature of the application, each of these senses will contribute differently to task performance, and task demands may conflict with optimal conditions for embodiment. In this section, we formulate three example tasks and discuss their links to embodiment subcomponents and the related research questions.

#### Social Interaction at a Distance Requiring a High-Level Ownership

A task related to teleoperation (that is not further elaborated in this paper) is social interaction with people in a remote environment. Applications that should provide a convincing and engaging experience of social presence (the sense of being with another sentient being or person: Biocca et al., 2003; e.g., in social telepresence or social robotics: Nakanishi et al., 2014; Tanaka et al., 2015) require a realistic (anthropomorphic) remote self-representation with a similar posture and sufficient spatial overlap with the corresponding real body, in combination with a 1PP (preferably stereoscopic) display and a high degree of visuotactile synchronicity (Oh et al., 2018; Ventre-Dominey et al., 2019). The mere presence of (and interaction with) people in a social setting may also affect the sense of embodiment. For instance, in a social conversation setting with a teleoprated android robot it was found that the operator's sense of embodiment is enhanced by the interaction with the social partner, while the partner's presence also enhances the sense of telepresence (Nishio et al., 2013). Although the findings reported in the previous sections indicate that embodiment can be induced for any type of object, a stronger sense of embodiment is typically achieved with fake body parts that resemble real ones. Open questions are for instance: What part of the human body (self) needs to be represented to create a convincing experience (Beckerle et al., 2018)? How, and to what extent, does embodiment in a mediated environment depend on the structure, morphology, topology, size and dynamics of the body's representation (Kilteni et al., 2012a; Ventre-Dominey et al., 2019)?

#### Remote Execution of Specialized Tasks Requiring a High Level of Agency

When high levels of agency are required (e.g., in applications like robotic surgery or explosive ordnance disposal; Nahavandi et al., 2015; Enayati et al., 2016; Pacchierotti et al., 2016), a teleoperation system should provide a close match between predicted (intended) movements and actual kinesthetic feedback (i.e., efficient and transparent motor control). Open questions are for instance: What are the limits of visuomotor congruency needed to establish agency? What are the effects of visual perspective on agency for a given task? What are the effects of delayed or interrupted control on agency? How can scaling of forces or dimensions be applied without reducing agency (too much)?

#### Reconnaissance in Dangerous Environments Requiring a High Level of Self-Location

When a high degree of situational awareness is required (e.g., in applications like reconnaissance or search-and-rescue operations in unpredictable or hazardous environments; Trevelyan et al., 2016), a 3PP might provide a better awareness of the environment compared to a 1PP (van Erp and Kappé, 1997). In that case, embodiment of a device seen from a 3PP requires additional reinforcement in the form of synchronous visuotactile information. Open questions are for instance: What type and how much synchronous visuotactile feedback is needed to establish embodiment of a telemanipulator seen from a 3PP? Is this feedback only required at the start of an operation to induce embodiment or is it required to maintain embodiment all through an entire operation? What is the influence of switching between 1PP and 3PP?

### Embodiment Limits for Non-corporeal Objects

An interesting question in the context of teleoperation is where the limit lies regarding the brain's capacity to integrate robotic tools as part of one's own body. It is known that there are limits to the discrepancies for which embodiment can still be induced, or in terms of the predictive encoding framework: if the prediction errors become too large, the brain will not explain the sensory input based on an embodiment model but will instead consider the tool as an external object. As mentioned before, an ownership illusion does not arise for fake body parts that have an unrealistic (e.g., a wooden stick (Tsakiris and Haggard, 2005) or abstract (Yuan and Steed, 2010) appearance, that have impossible postures (Ehrsson et al., 2004; Tsakiris and Haggard, 2005; Costantini and Haggard, 2007) or that are located outside the peripersonal space (Lloyd, 2007). An interesting question is to what extent such an object can evoke embodiment when the brain is given a chance to gradually get accustomed to the object and slowly reduce its prediction errors, for instance through morphing or other visual manipulations.

### Long Term Effects

Also, not much is known about the long-term effects of embodiment. When participants were trained for 3 days in a RHI set-up, no long-term effect was found on ownership, although the training seemed to facilitate multisensory integration, reflected in a proprioceptive drift (Honma et al., 2014). It has also been suggested that sleep can serve as a “reset” for learning embodiment. This could be an important factor for non-realistic remapping, e.g., embodiment involved in remapping of existing body part relations, such as a remapping of the right thumb to a virtual right arm (Kondo et al., 2018b). Children are as sensitive to visuotactile synchrony as a source for the rubber hand illusion as adults, but exhibit a larger proprioceptive drift, indicating that several different processes, with different speeds of development, underlie the sense of embodiment (Cowie et al., 2013). For hand prostheses, it has been shown that daily use leads to a stronger response in visual hand-selective areas in the brain when presented with images of hand prostheses, even when these do not resemble a human hand (e.g., hooks; van den Heiligenberg et al., 2018). This suggests that frequent embodiment leads to neurophysiological changes, the implications of which are not fully known yet.

### Individual Differences

More research is needed to understand the physiological mechanisms and personality factors (e.g., susceptibility, immersive tendencies) driving embodiment. The response to the RHI is known to vary greatly across individuals: some people are more prone to embodiment than others (Lin and Jörg, 2016). Even with perfect visuotactile or visuomotor synchronicity, the feeling of ownership over a fake body part occurs in only about 70% of the population, while the other 30% does not experience ownership (Ehrsson et al., 2005a; Lloyd, 2007; Martini et al., 2014). In addition to sensory integration, it appears that personality factors also modulate embodiment: people with higher cognitive flexibility (Yeh et al., 2017) or higher emotional intelligence (Perepelkina et al., 2017) have a stronger ownership experience.

### Testing the Predictive Encoding Theory as Useful Framework

We have used the predictive encoding theory as framework for the interpretation and discussion of a range of embodiment results. The same theory can be used to make predictions on embodiment effects that are of interest in telerobotics. For instance, the prediction that once the embodiment model is activated, gradual changes will lead to adaptation of the model and will not break the embodiment illusion. This leads to the prediction that a slow “morphing” from one's own arm into any robotic tool as well as to superhuman morphologies is possible, as long as the change is gradual. The “optimal” speed and increment of changes have yet to be determined.

### The Ultimate Assessment of the Weight of Different Sensory Cues

As we showed in section Premise 1: The Body Representation Is Malleable and Can Include Non-bodily Objects, many sensorimotor cues contribute to embodiment, including visuotactile synchrony and realism, visuomotor and proprioceptive synchrony, visual perspective, visual realism. But how do these factors quantitatively compare in evoking embodiment? Most studies investigate only one or two of these factors, which makes it possible to conclude that all can contribute but none of them is strictly required. However, it has neither been investigated how the brain weights the different cues, nor what the boundary conditions are. Determining the weights and possible interactions between cues requires a single elaborate experiment.

### The Ultimate Assessment of Embodiment Causing Performance Improvement

Only few studies have investigated the relation between embodiment and task performance and as far as we are aware only in the domain of prostheses but not in telerobotics. The ultimate test of our theorem is to explicitly link (the degree of) embodiment with (the degree of) dexterous task performance. This can for instance be done by measuring both dexterous performance and sense of embodiment for an operator performing a teleoperation task. Measuring both entities on an interval scale will also yield more insight in the nature of their relation (e.g., whether it is linear or stepwise). Catoire et al. (2018) presented a set of simple and generic standardized benchmark dexterity tests that are representative for a broad range of inspection, maintenance, and repair activities and that can be used for the assessment of dexterous performance in teleoperation settings.

### Work Toward a Standardized Methodology and Effect Measures

Since Botvinick and Cohen first reported their studies on the RHI, a plethora of research using the same or very similar paradigm has been reported (Botvinick and Cohen, 1998; Lenggenhager et al., 2007; Kilteni et al., 2012b). However, over the last decade, many new paradigms and measures were introduced to study embodiment, for instance using new VR techniques. Also, some of the different subcomponents of embodiment are used haphazardly and new constructs are introduced without clear definitions. To mature, the field needs rigorous replication, methodologically well-designed studies and protocols, and a limited number of effect measures to enable comparisons across studies. This pleads for defining a uniform set of validated and standardized measures that covers the different subcomponents of embodiment (Riemer et al., 2019). This set could include basic physiological measures, subjective measures, and proxies such as proprioceptive drift.

### Countering System Limitations in Telerobotics

To afford a convincing multisensory experience a telerobotics system should provide (1) the synchronicity (match) between the different (visual, auditory, and tactile) sensory signals that is required to achieve a coherent multisensory experience and (2) the bandwidth required to optimally convey relevant cues. The coding of video and tactile data should be sufficiently efficient, and system-intrinsic effects such as time delays, jitter, or packet loss should be minimized to maintain the levels of system transparency and stability that are required to provide an immersive and synchronous multisensory experience. This is especially the case for tactile information channels, since tactile information is transmitted bidirectionally due to the law of action and reaction, unlike visual and auditory information. Time delays (which inevitably occur when remotely communicating haptic information) will seriously degrade this bidirectional flow of information. Additional problems occur when these disturbances induce discomfort or fatigue. Even a small amount of discomfort may become unacceptable when users need to operate telerobotic devices over longer periods of time.

Agency and ownership can serve to alleviate the temporal constraints for multisensory integration (Maselli et al., 2016). Once agency and ownership have been established, people fail to notice asynchronies in visuotactile stimulation that are otherwise (in the absence of the illusion) detected (Maselli and Slater, 2013). This suggests including an ownership induction phase in the use of telerobotic systems. This can for instance be achieved through (dynamic or static) active self-touch, which effectively elicits a sense of ownership (Hara et al., 2016). It has also been suggested to deploy affective feedback to integrate teleoperation devices into the bodily self-representation (Beckerle et al., 2018). Affective touch (e.g., slow stroking) effectively induces a strong sense of ownership (Haans et al., 2012; Crucianelli et al., 2013, 2018; Lloyd et al., 2013; van Stralen et al., 2014; de Jong et al., 2017). Finally, bi-directional human-machine interfaces may serve to enhance ownership and agency by integrating intent detection and multi-sensory feedback through co-adaptation (the process through which the robotic device adapts to its human operator/user while the human adapts to the device: Beckerle et al., 2019).

Another way to counter system limitations and facilitate embodiment could be previous training in optimal conditions (zero delay, sufficient bandwidth), since the memory of past agency significantly contributes to the sense of embodiment (Liepelt et al., 2017) and because training facilitates multisensory integration (Honma et al., 2014).

As for the relative contribution of visuotactile and visuomotor feedback to the body ownership illusion, it appears that synchrony in one kind of feedback compensates for asynchrony in the other modality (Huynh et al., 2019).

## Conclusions

We postulate that the sense of embodiment of a remote manipulator improves dexterous performance in a teleoperation context. We reasoned along four premises: (1) The representation of the body in the brain is malleable and can include non-bodily objects, (2) Embodiment can be elicited through mediated (or even virtual) sensorimotor interaction, (3) Embodiment has a degree of robustness against inconsistencies in appearance, size, movement, etc. of the embodied object, and (4) Embodiment improves performance. We showed that each of these premises is substantiated by results reported in the literature (with the possible exception of the last one, which is at least shown for the use of prostheses; see Table 2). However, there is currently no evidence that directly links embodiment and teleoperation performance.

Assuming this direct link exists, guidelines are needed on how to increase transparency in telerobotics by implementing an embodiment approach in addition to current technical performance measures. This approach should take heed of the three components of embodiment: agency, ownership and self-location. Based on the supportive evidence for our second premise (“*embodiment can be evoked by mediated sensorimotor interaction*”) that was presented in section Premise 1: The Body Representation Is Malleable and Can Include Non-bodily Objects, we now formulate the following set of initial guidelines: teleoperation systems should provide (1) a convincing 1PP to achieve a high degree of self-location (section Visual Perspective: A 1PP generally yields a stronger embodiment in terms of self-location and ownership), (2) a close match between predicted movements and actual kinesthetic feedback to induce high levels of agency (section Visuomotor and Proprioceptive Cues: visuomotor and proprioceptive synchrony significantly induce agency), (3) a spatiotemporal match between felt and seen haptic stimuli (section Visuotactile Stimulation: visuotactile synchrony significantly induces embodiment), and (4) a sufficient visual likeness to the human body to induce high levels of embodiment (section Visual Realism (Similarity): visual likeness helps to induce embodiment). The relative importance of these factors may change per context and task.

We adopted the predictive encoding theory as a framework to interpret and discuss the importance of multisensory cues for the strength of embodiment. This leads to the following hypotheses: (1) More sensory cues are more effective provided they match the predictions of an embodiment model; (2) Errors in a modality do not automatically lead to a lack of embodiment, and none of the senses is strictly required for embodiment to occur; (3) Sudden large prediction errors may stop the activation of the embodiment model, but: (4) The brain will cope with smaller prediction errors by updating the embodiment model. This suggests that a flexible embodiment model can be achieved, by slowly introducing prediction errors, thus allowing the brain to gradually adjust its embodiment model.

## Author Contributions

JE conceived the idea for this study. JE and AT performed the literature study and wrote the draft manuscript. All authors were actively involved in the revisions of the original drafts and in writing the final version.

### Conflict of Interest

The authors declare that the research was conducted in the absence of any commercial or financial relationships that could be construed as a potential conflict of interest.
